# Investigating expert performance when observing magic effects

**DOI:** 10.1038/s41598-022-09161-5

**Published:** 2022-03-24

**Authors:** Elias Garcia-Pelegrin, Clive Wilkins, Nicola S. Clayton

**Affiliations:** Department of Psychology, University of Cambridge, Cambridge, UK

**Keywords:** Psychology, Human behaviour

## Abstract

The use of magic effects to investigate the blind spots in attention and perception and roadblocks in the cognition of the spectator has yielded thought-provoking results elucidating how these techniques operate. However, little is known about the interplay between experience practising magic and being deceived by magic effects. In this study, we performed two common sleight of hand effects and their real transfer counterparts to non-magicians, and to magicians with a diverse range of experience practising magic. Although, as a group, magicians identified the sleights of hand as deceptive actions significantly more than non-magicians; this ability was only evidenced in magicians with more than 5 years in the craft. However, unlike the rest of the participants, experienced magicians had difficulty correctly pinpointing the location of the coin in one of the real transfers presented. We hypothesise that this might be due to the inherent ambiguity of this transfer, in which, contrary to the other real transfer performed, no clear perceptive clue is given about the location of the coin. We suggest that extensive time practising magic might have primed experienced magicians to anticipate foul play when observing ambiguous movements, even when the actions observed are genuine.

## Introduction

Magicians use intricate techniques of deception that capitalise on the spectator’s blind spots in attention and perception and roadblocks in cognition^1–3^. The interest in how magic effects exploit such psychological constraints has been growing recently within the psychological community, and more frequently new evidence regarding the qualities that make the observer liable to these techniques is being unearthed. For example, when observing sleight of hand, curved motions appear to have a larger effect in misdirecting the observer than utilising rectilinear motions^4^. While the use of social misdirection techniques such as joint attention might strengthen a magic effect^5^, these techniques do not seem to be imperative for their success^6,7^. Although the investigation of magic effects has produced thought-provoking results on how the techniques operate on the spectator in both humans and non-human animals^8^, the investigation of how practising magic affects magicians’ perceptual and cognitive abilities merits further exploration.

To become proficient at reliably fooling their audience, most magicians spend years training in the craft through meticulous deliberate practice^9,10^. Attaining a high level as a magician is not an easy task, for example out of an average of 1500 professional magicians at The Magic Circle© only 20% of them have reached the highest rank (Gold star). Magicians’ extensive training in sleight of hand grants them very specific motor-performance skills^11,12^, which allows them to seemly mislead their spectators into experiencing the impossible. Indeed, it is these abilities that make them experts at manipulating their audience’s perception and attention^13,14^. Consequently, one might justifiably consider the study of magicians to inherently be the study of expertise.

Experts (i.e., those who, through practice, have perfected a particular domain) have been the topic of interest of scientists for centuries^15,16^, and some have aimed to disentangle whether expertise in a particular domain is a transferable skill and therefore granted the experts with superior ability in a different array of perceptual tasks^15,17,18^. Research investigating the abilities gained through expertise across different domains has yielded significant results in many areas of expertise such as chess^19,20^, music^21–23^, sport^24–27^, and even medicine^28^. It is currently well established that while experts elicit superior performance over novices in domain-related tasks, they do not tend to significantly differ outside of the construct of the domain in which they are experts in^28–30^. For example, when testing experienced handball goalkeepers and novices in a reaction time task that was directly related to handball goalkeeping (moving the right or left hand towards a target in a handball goal), the experienced goalkeepers demonstrated a superior perceptual and reactionary ability in comparison to the novices. However, when tested in a similar yet not specific to goalkeeping task, the expert group did not show a significant effect of expertise in their reaction times when compared to novices^31^. The occurrence that experts gain superior perceptual abilities concerning the domain they regularly practice is understandable. The close link between action and perception is hardly up for debate^32–34^, and this relationship is further pronounced in the role of expertise, as expert behaviours are mostly directed towards specific targets. As such, perceptual mechanisms in experts tend to adapt to the niches of the action patterns that are performed regularly^35^. The evidence that learning in some domains can affect the visual recognition of specialized patterns is evidenced in dentistry^36^, chemistry^37^, meteorology^38^, and geology^39^ to name but a few examples. The deliberate practice theorem suggests that expertise in a particular topic is developed and moderated through the amount of years purposely engaged in the training of the topic^15,28,35^. A classic example is chess, in which at least 10 years of intense and regular practice is necessary to become a master^20^. However, little is known regarding the intricacies behind the amount of deliberate practice necessary to develop expert faculties, whether the pattern holds true for other modalities, and in what way the amount of deliberate practice in one domain alters the expert’s perception in contrast to novices, or those of other expert’s that have less experience.

Therefore, one might pose the question of whether magicians, whose expertise involves being highly adept at prestigitation, namely object manipulation and action mimicry, display domain-specific expertise in such areas when compared to non-magicians, and whether this expertise is moderated by the amount of experience, for example as measured by the number of years perfecting the craft. Evidence from expertise studies in magicians reveals that magicians appear to excel in comparison to non-magicians in several faculties. For example, when observing video recordings of people performing reach to grasp actions, sleight of hand practitioners evidenced an enhanced ability to discriminate real from pantomimed actions when compared to a sample of non-magicians^40^. Moreover, in comparison to non-magicians, magic practitioners appear to excel at producing pantomimed grasping actions of objects (but only when the said object is being observed while producing the pantomime)^41^ and appear to have a more sophisticated mental hand representation^42^. Consequently, the laborious training in sleight of hand that magicians undergo seems to grant their perpetrators an advantage when observing and performing manual manoeuvres. However, little is known about the interplay between the blind spots in perception exploited by sleight of hand effects and their relationship with magicians’ expertise. Sleight of hand techniques are particularly powerful as they capitalise on the spectators’ expectations of typical object exchanges between hands. Manipulations of objects between hands are typically learned early in development, performed regularly, and seldom pondered upon once mastered^43^. Similarly, when observing others moving objects between hands, we use our previous experience of object manipulation and observation of others manipulating to assume the most likely outcome (i.e., that the object has been transferred successfully), thus completing the movement even when we cannot explicitly see it (i.e., amodal completion^44,45^), and, when observing sleight of hand, missing the potential cues that might reveal foul play^46,47^.

**Figure 1:**
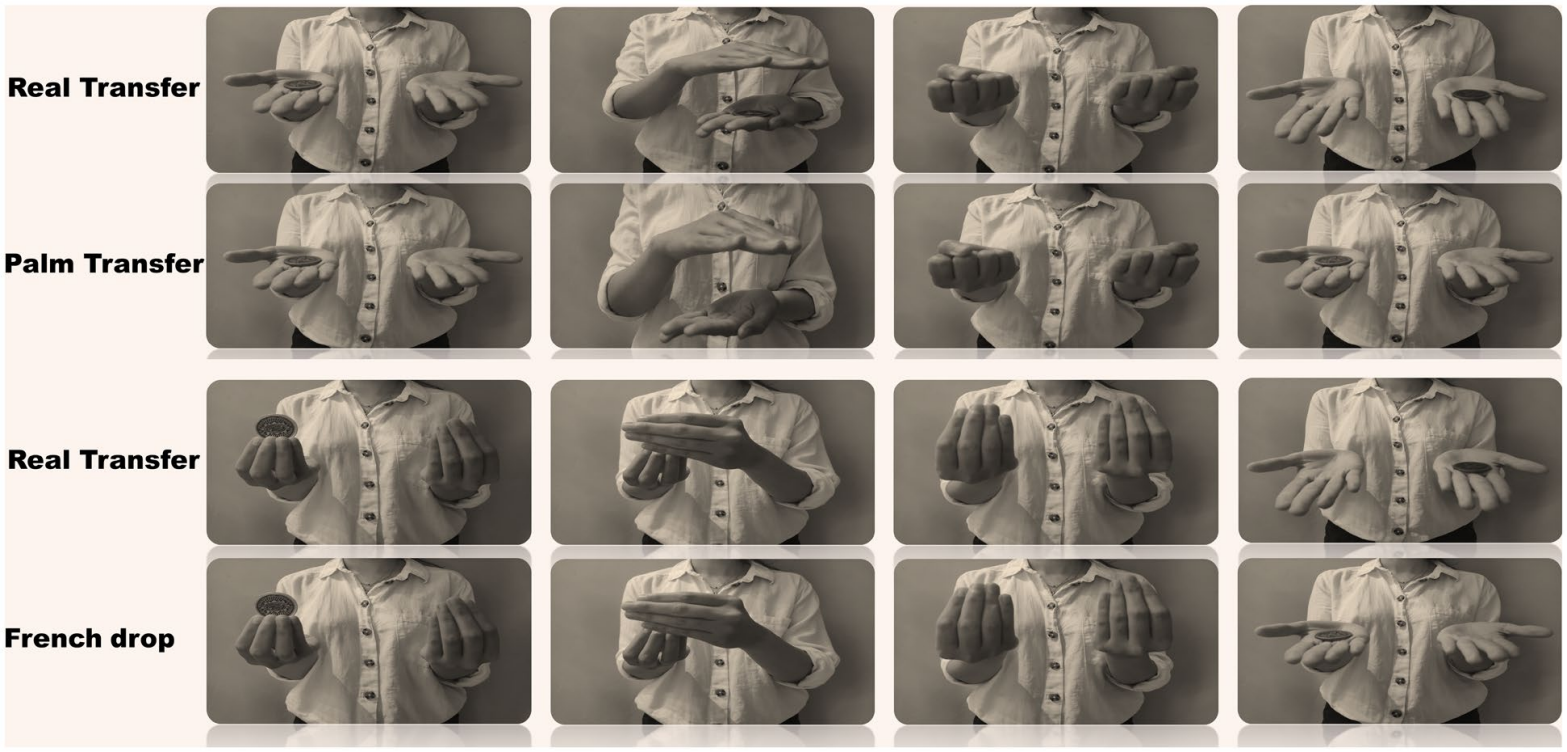
Hand movements of the magic effects (Palm transfer and French drop) and corresponding real transfer counterparts.

In this study, we investigated whether magicians were more proficient at identifying sleight of hand movements from real transfers of objects than non-magicians. We performed two common sleight of hand techniques and their real transfer counterparts to a sample of 165 non-magicians, and a sample of 93 magicians with different expertise levels. The sleight of hand techniques used (palming and French drop) are typically employed to mislead the spectators into believing one object has successfully been transferred from one hand to the other, while in truth the object remains concealed in the initial hand. These are common methodologies used in most close-up magic routines and typically learnt early on when studying sleight of hand. While similar in its importance for a good grasp of magic performance, both palming and French drop techniques differ greatly in the visual cues given to the observer. Palming involves the retention of an object between the palmar muscles of the hand whilst simultaneously mimicking the transfer of the object from one hand to the other. In contrast, the version of the French drop used in this study involves mimicking the grab of the object with one hand, whilst simultaneously dropping the object so that it falls out of the hand that was holding it (Fig. 1). Given the plethora of evidence suggesting that experts in one domain tend to excel at perceptive tasks which are related to such domain, we hypothesise that magicians will outperform non-magicians at differentiating real from sleight of hand transfer.

## Methods

The choices of the 165 non-magicians (mean age = 37, 70 males) were obtained prior to this study for a different experiment that involved the participants observing three magic effects (Palming, French drop, and Fast Pass) and a subsequent comparison with the choices of a sample of Eurasian jays (*Garrulus glandarius*) (see “Supplementary Information S1” in^8^. The sample of magicians (n = 93) was recruited from The Magic Circle©, the Leicester Magic Circle, and the Cambridge University Magic Society social media platforms and all of these participants were provided with access to the survey via a link. The sample consisted of magicians with a diverse amount of experience practicing magic (1–4 years (n = 28, mean age = 23, 15 males); 5–10 years (n = 35, mean age = 30, 31 males); and > 10 years (n = 30, mean age = 45, 29 males). Sample sizes where determined through calculations provided by G*Power per sample size^48^. Both magicians’ and non-magicians’ surveys contained the exact same videos of the French drop and palming magic effects and real transfer counterparts. Participants in the non-magician survey observed an extra magic effect and an extra control condition per effect, which were performed specifically for the comparison with non-human animals, thus being irrelevant to the purpose of this study and omitted for the magician survey. Both surveys were designed using Qualtrics, the magician survey consisted of 4 videos for each of the two experiments (i.e., 2 videos per magic effect, 2 videos per real transfer counterpart, each one being performed right to left, and vice versa), the non-magician survey consisted of the same videos and arrangement plus the extra unrelated magic effect and control conditions. The videos of the effects consisted of purposely pre-recorded videos of the experimenter’s hands (30 year-old male with 12 years of experience as a magician) performing the effects, the videos were presented full screen at 30 fps, the experimenter had an **O** (left hand), or **X** (right hand) painted on the back of each corresponding hand for better identification by the participant. Participants were told they were participating in a human perception study and were asked to attentively observe each video of the magic effect and then identify which hand contained the coin by choosing **O** or **X** appropriately. The order of the videos was randomised for each candidate. All participants were provided with the relevant information of the study and gave their informed consent prior to their participation. This study was not preregistered.

### Ethical approval

As the experiment did not involve the subjects disclosing any information about their individual identity, the participants were not from any vulnerable group, and the conditions of the experiment did not pose any risk to the participant, the experiment did not require ethical approval by the University of Cambridge. All procedures were performed in accordance with relevant guidelines.

### Conditions

#### Palm transfer

In this condition, the experimenter utilised a palming technique to simulate a coin being transferred from one hand to the other. The technique involves the concealment of the coin in the middle of the palm, held together by loosely clenching both the right and left sides of the palm (i.e., the thenar and hypothenar muscles). When properly performed, the magician takes an object with one hand, secures it in their palm, and then fake transfers it to the other hand whilst simultaneously pretending to catch the object, even though in reality, the object remains in the original hand. To the naïve spectator, however, it will appear to be a seamless transfer of objects from one hand to the other.

#### Palm real transfer counterpart

In the real transfer condition for this experiment, the coin was genuinely transferred from one hand to the other. The experimenter recreated the same movements that were used in a Palm transfer but allowed the coin to drop to the opposing hand, instead of retaining it in the palm. Note that in contrast with the magic effect version, in this transfer the coin could be seen being dropped from one hand to the other.

#### French drop

The French drop condition also relied on another fake transfer technique used by magicians. This method of sleight of hand consists of holding a coin in one hand and then mimicking a grabbing motion of the coin that is held between the thumb and both the index and middle finger of the opposite hand, creating the illusion that the coin has been transferred from one hand to the other. This sleight utilises the hand mimicking the grab of the coin to occlude how the coin is dropped inside the palmar region of the original hand holding it, while simultaneously mimicking how the grasping hand enacts a precision grip on the same coin.

#### French drop’s real transfer counterpart

The French drop’s real transfer condition simulated the same movements as the French drop condition without allowing the coin to drop to the palm of the hand holding it. Thus, the coin was grabbed by the hand performing the precision grip and genuinely transferred from one hand to the other. In order to keep both movements as similar as possible, the coin was still occluded by the grasping hand when grabbed. Note that in neither of the conditions of this experiment did the coin appear in view after being occluded by the grasping hand.

### Analysis

The data was analysed using JASP (v.0.10.3, http://jasp-stats.org) and RStudio for Mac (version 1.2.1335). To determine whether there was a significant difference between correct and incorrect choices per condition in each group we used binomial tests (against value: 0.5). To investigate the effects of experience and condition as explanatory variables we performed generalized linear mixed models (GLMM) with subject specified as a random term in the model to control for repeated measures^49^. Significant differences between treatments were further explored using post-hoc pairwise comparisons and were adjusted using the Holm-Bonferroni method to maintain the overall alpha level at the nominated value or 0.05 for multiple pairwise comparisons. All data, unique materials, video conditions, documentation, and code used in the analysis can be found at the Open Science Framework database at: https://osf.io/vd82u/?view_only=cd7fd55cf942476bbb825f4a7cff0693

## Results

### Palm transfer

#### Non-magicians versus magicians

**Figure 2:**
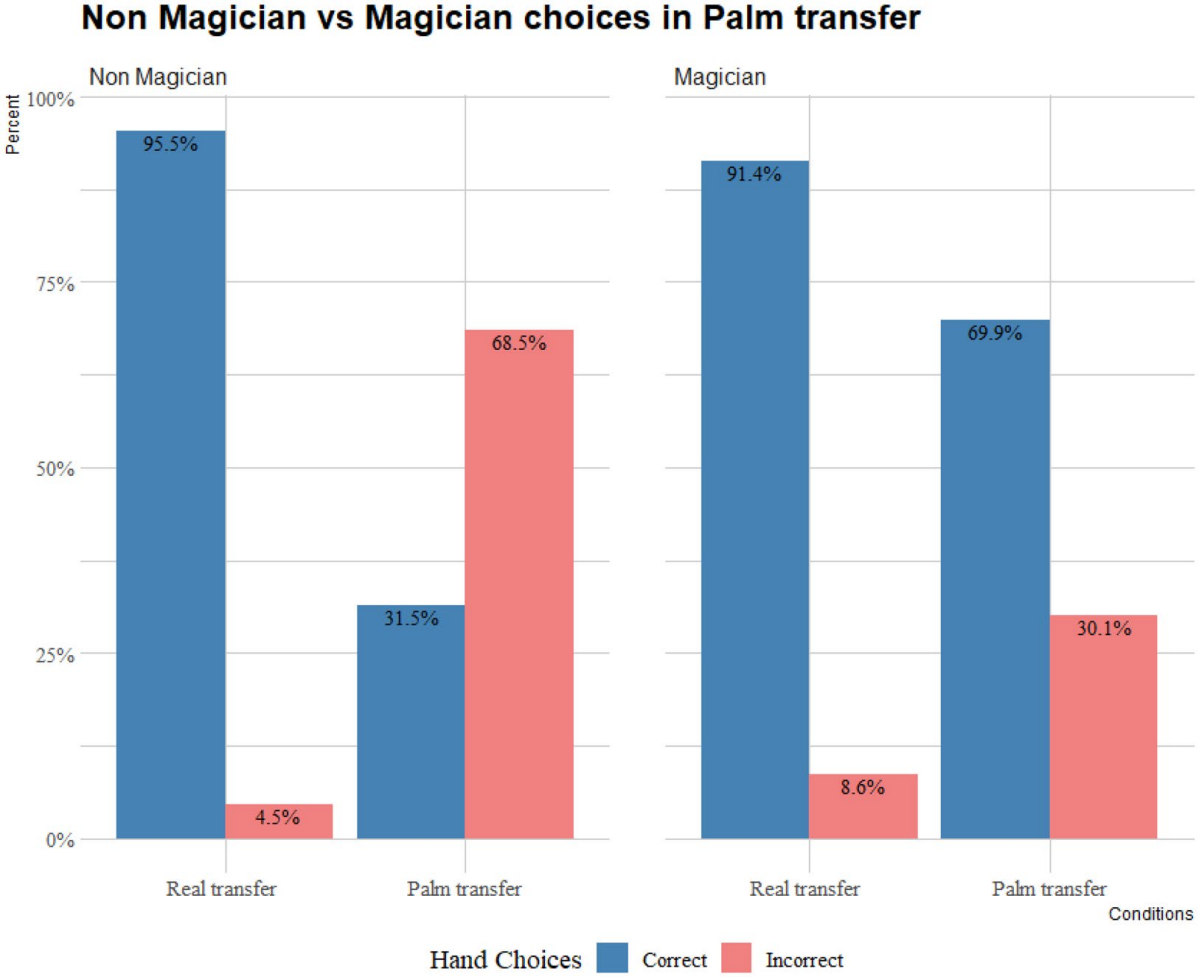
Choices of non-magicians and magicians in palm transfer and real transfer counterpart.

As shown in Fig. 2, both non-magicians and magicians significantly chose the correct hand when observing the real transfer counterpart (*binomial test: p* < 0.001 for both*)*. However, only magicians significantly chose the correct hand when observing a palm transfer (*binomial test*: *p* < 0.001), while non-magicians significantly choose incorrectly (*binomial test*: *p* < 0.001) i.e., non-magicians were significantly misled by the motions of the magic effect, whereas the magicians were not.

A GLMM revealed that there was a significant effect on the participants’ choices for condition (χ^2^ = 135.695; df = 1; *p* < 0.001), significant effect for skill (i.e., non-magician or magician) (χ^2^ = 26.134; df = 1; *p* < 0.001), and significant interaction between condition and skill (χ^2^ = 31.701; df = 1; *p* < 0.001). Post-hoc pairwise comparisons with the Holm-Bonferroni adjustment showed that both non magicians and magicians’ choices in the real transfer significantly differed from their respective choices in the palm transfer (*p* < 0.001 for both). Non-magicians and magicians’ choices did not significantly differ in the real transfer (*p* = 0.07), but they did so in the palm transfer (*p* < 0.001).

#### Effect of experience practising magic

**Figure 3:**
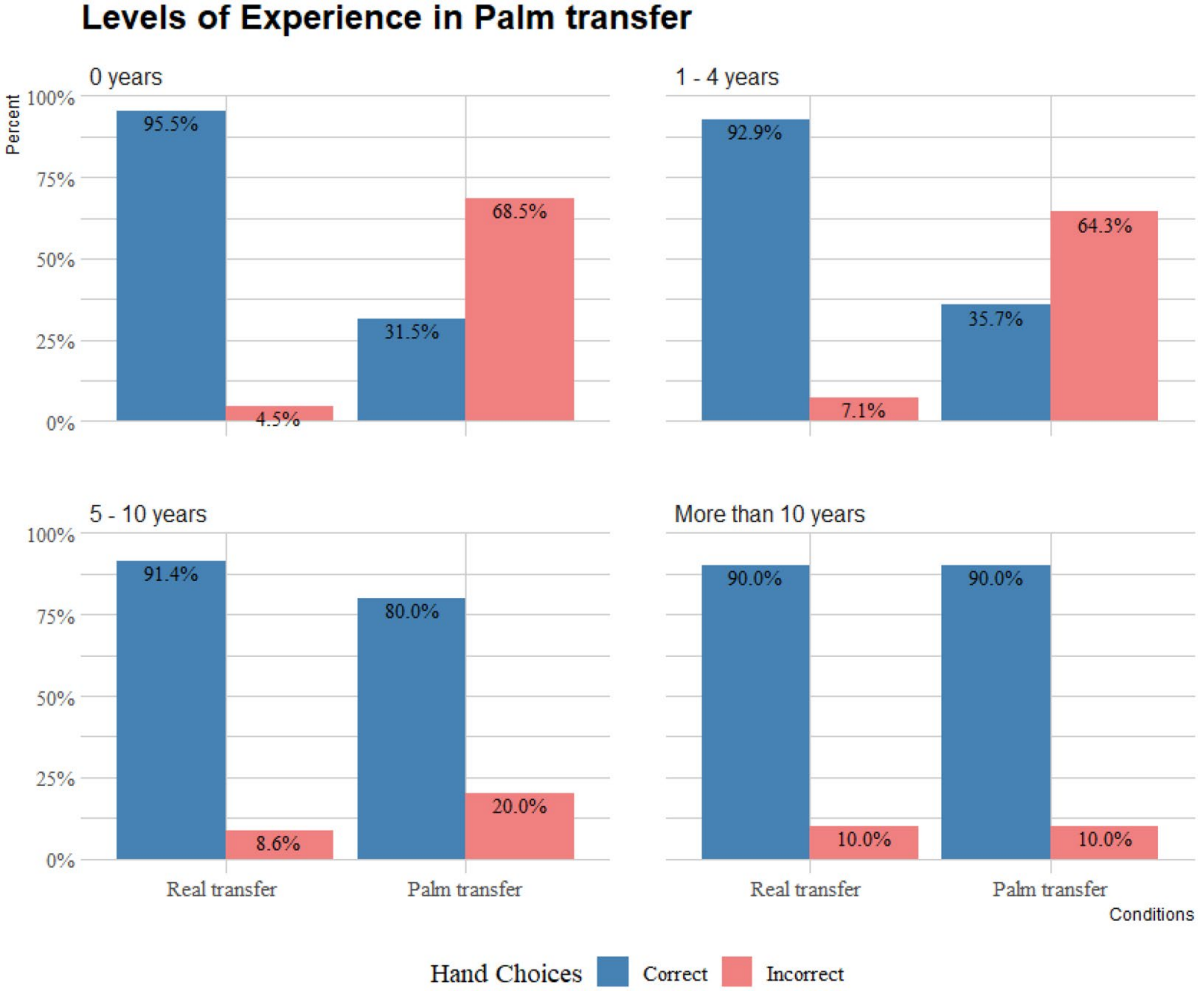
Choices in palm transfer, and real transfer counterpart separated by levels of experience practicing magic.

In order to investigate the effects of experience on the participants’ choices, the magician’s group was split into three levels of experience (1–4 years (n = 28, mean age = 23, 15 males); 5–10 years (n = 35, mean age = 30, 31 males); and > 10 years (n = 30, mean age = 45, 29 males). Binomial tests showed that all participants were more likely to choose the correct hand when observing a real transfer independently of their level of experience performing magic (*binomial test*: *p* < 0.001). When observing a palm transfer, however, non-magicians, and magicians with 1–4 years of experience significantly chose incorrectly (*binomial test: p* < 0.001, and *p* = 0.044 respectively). By contrast, magicians with 5–10 years of experience and more than 10 years of experience significantly chose correctly (*binomial test: p* < 0.001) (Fig. 3).

The GLMM showed that there was a significant effect of condition (χ^2^ = 118.347; df = 1; p < 0.001), a significant effect of experience practicing magic (χ^2^ = 26.210; df = 3; *p* < 0.001), and a significant interaction between condition and experience (χ^2^ = 46.508; df = 3; *p* < 0.001). To investigate this further a series of post-hoc pairwise comparisons with the Holm–Bonferroni adjustment were performed. The choices of all magicians significantly differed between their choices in the real transfer and their respective choices in the palm transfer (*p* < 0.001 for all). There was no significant difference in the palm transfer condition between non-magicians and magicians with 1–4 years of experience (*p* = 1), and no significant difference between magicians with 5–10 years of experience and magicians with more than 10 years (*p* = 1). When observing a palm transfer, both the choices of magicians with 5–10 years of experience and magicians with more than 10 years significantly differed from non-magicians’ choices (*p* < 0.001 for both), and the choices of magicians with 1–4 years of experience (*p* < 0.001 for both). There was no significant difference in the real transfer condition between non magicians and magicians irrespective of their level of experience performing magic.

### French drop

#### Non-magicians versus magicians

**Figure 4:**
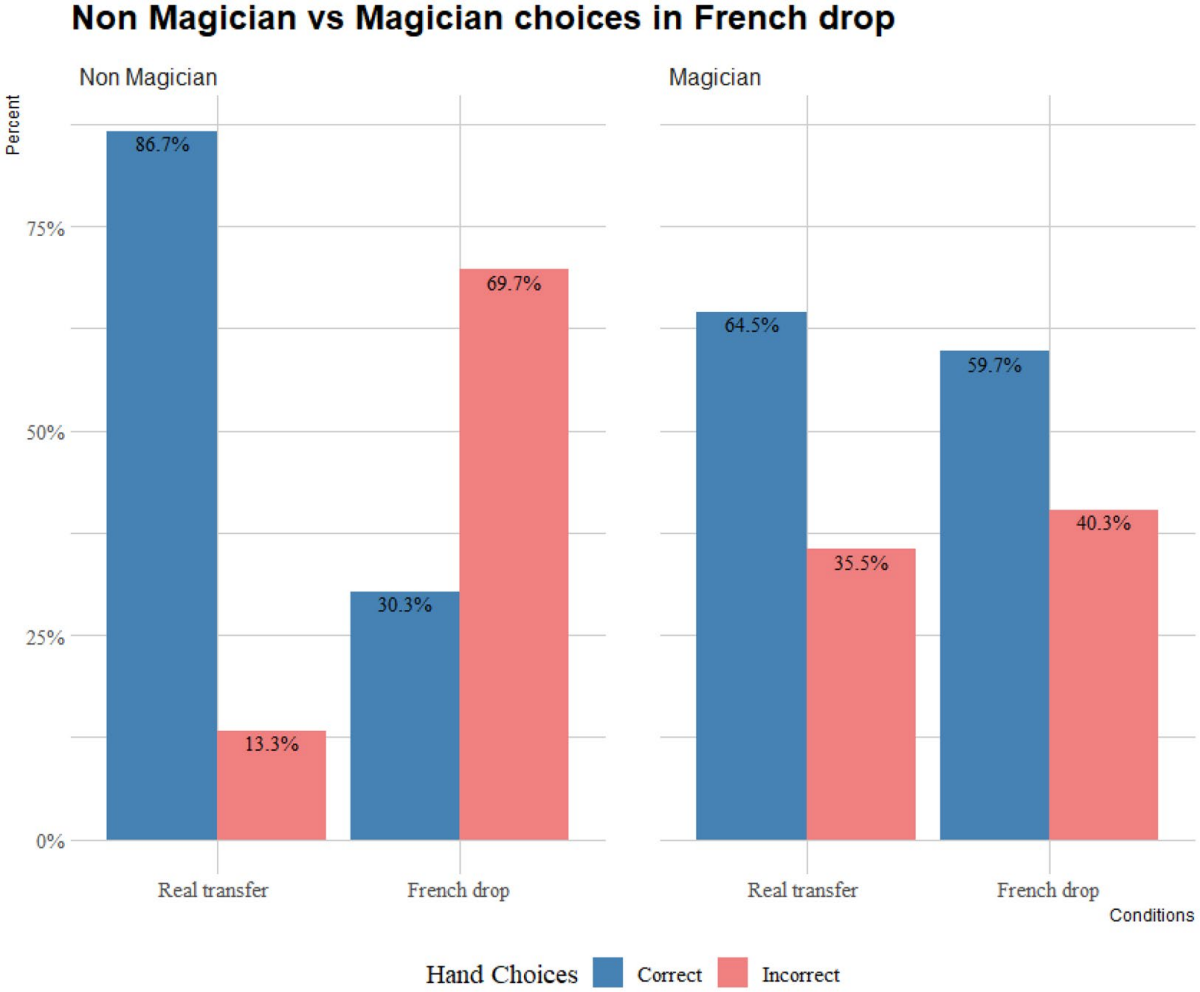
Choices of Non-magicians and magicians in French drop and real transfer counterpart.

Figure 4 shows the choices for both non-magicians and magicians in the French drop, in which both non-magicians and magicians significantly choose correctly when observing a real transfer (*binomial test*: *p* < 0.001 for both). However, non-magicians significantly chose incorrectly when observing a French drop (*binomial test*: *p* < 0.001), while magicians significantly chose correctly (*binomial test*: *p* = 0.01).

The GLMM showed a significant effect in the participants’ choices for condition (χ^2^ = 108.9570; df = 1; *p* < 0.001), but not for skill (χ^2^ = 1.2709; df = 1; *p* = 0.25), and a significant interaction between condition and level (χ^2^ = 72.2841; df = 1; *p* < 0.001). Non magician’s choices significantly differed between the real transfer and French drop (*p* < 0.001). There was no significant difference between the magicians’ choices in the real transfer and French drop (*p* < 0.33). There was a significant difference between non-magicians’ and magicians’ choices in the real transfer (*p* < 0.001) and their choices in the French drop (*p* < 0.001).

#### Effect of experience practising magic

**Figure 5:**
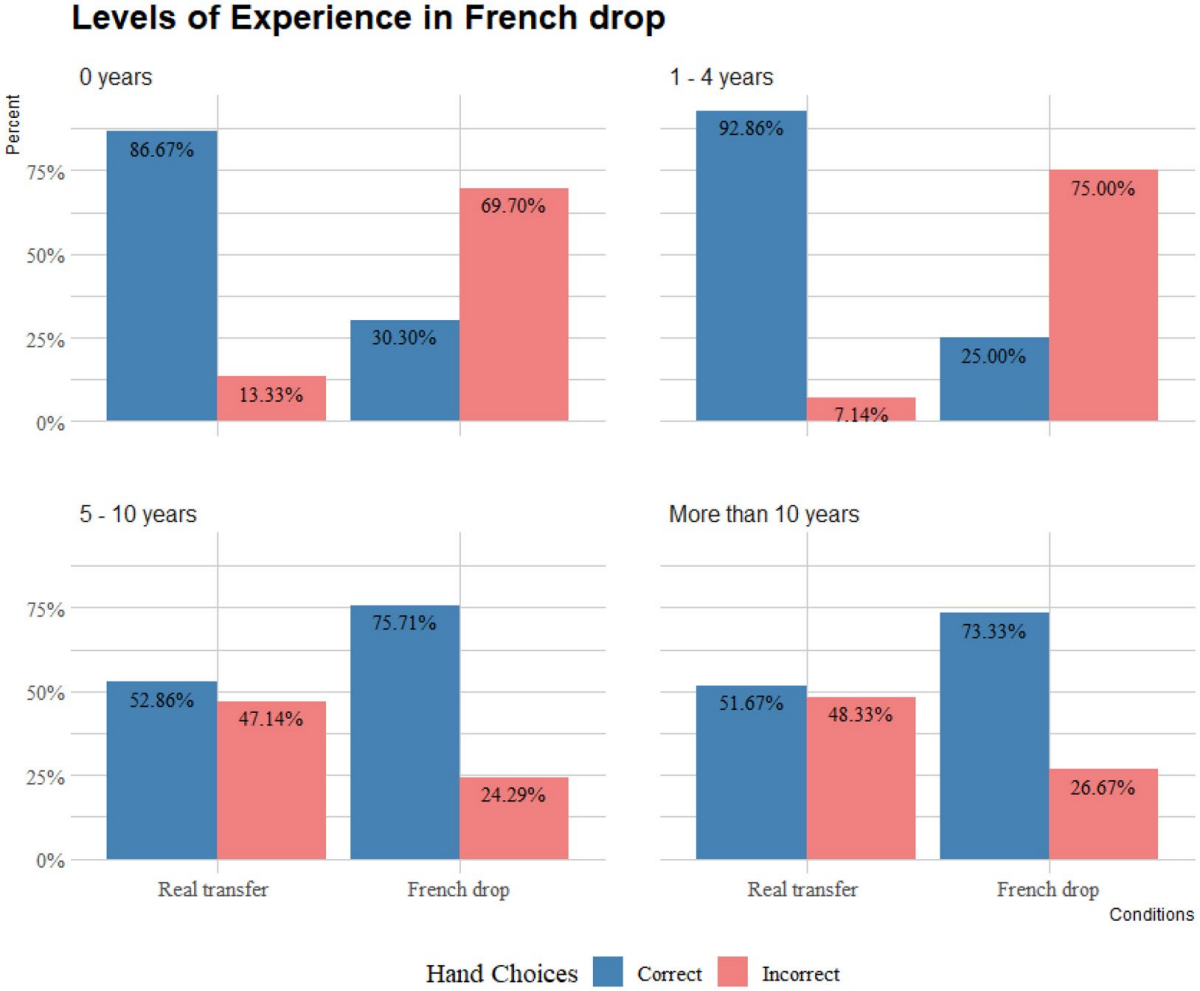
Choices in French drop, and real transfer counterpart separated by levels of experience practicing magic.

Similarly to the Palm transfer, to investigate the effect of experience practising magic in the participants’ choices, the magician’s group was split into their levels of experience. As shown in Fig. 5, when observing the real transfer, non-magicians, and magicians with 1–4 years of experience were more likely to choose the correct hand (*binomial test: p* < 0.001 and *p* = 0.005 respectively). There was no significant difference in the choices of magicians with 5–10 years of experience (*binomial test: p* = 0.72), and more than 10 years of experience (*binomial test: p* = 0.89). Magicians with 1–4 years of experience were more likely to choose incorrectly when observing a French drop (*binomial test: p* < 0.001 for both). Conversely, magicians with more experience were more likely to choose correctly (5–10 years of experience and magicians with more than 10 years of experience (*binomial test*: *p* < 0.001).

The GLMM revealed that there was a significant effect in the participants’ choices for condition (χ^2^ = 95.6946; df = 1; *p* < 0.001), and a significant interaction between condition and experience (χ^2^ = 135.0624; df = 3; *p* < 0.001). Binomial tests were conducted to investigate the number of correct vs incorrect choices per group, and a series of post-hoc pairwise comparisons with the Holm-Bonferroni adjustment were performed. Magicians’ with 1–4 years of experience choices significantly differed between the real transfer and French drop (*p* < 0.001). Magicians’ with 5–10 years of experience and magicians’ with more than 10 years of experience choices did not significantly differ between the real transfer and French drop (*p* = 0.059 and *p* = 0.12, respectively ).

There was no significant difference in the real transfer condition between non magicians and magicians with 1–4 years of experience (*p* = 0.8), and there was no significant difference in the real transfer condition between magicians with 5–10 years of experience and magicians with more than 10 years of experience (*p* = 1). However, the choices of both magicians with 5–10 years of experience and magicians with more than 10 years significantly differed from non-magicians’ choices (*p* < 0.001 for both), and magicians with 1–4 years of experience (*p* < 0.001 for both). There was no significant difference in the French drop condition between non-magicians and magicians with 1–4 years of experience (*p* = 1), and no significant difference between magicians with 5–10 years of experience and magicians with more than 10 years of experience (*p* = 1). Both the choices of magicians with 5–10 years of experience and magicians with more than 10 years significantly differed from non-magicians’ choices (*p* < 0.001 for both), and magicians with 1–4 years of experience (*p* < 0.001 for both).

## Discussion

In this study, magicians were better than non-magicians at differentiating real from fake transfers using both the palm transfer and the French drop. Furthermore, there was a clear relationship between the years of experience practising magic and the ability to detect sleight of hand effects. As expected, spectators of magic effects with no prior engagement in the craft were not able to differentiate a fake transfer from a real one in either experiment.

There was also a clear effect of expertise. The choices made by magicians with a limited amount of experience (i.e., 1–4 years of experience practising) did not significantly differ from the choices of non-magicians. By contrast, magicians with more than 5 years of experience practising magic excelled at not being misled by the sleight of hand techniques presented. Remarkably, while both participants with none or little experience and experienced magicians excelled at choosing correctly when observing the real transfer emulating the palming technique, only participants with none or little experience did so when observing a real transfer emulating the French drop movements. In this case, experienced magicians appeared to have difficulties at correctly identifying the hand containing the coin.

While, as a group, magicians in the Palm transfer performed significantly better than non-magicians at identifying a magic effect, further inspections of the magician’s experience revealed that only magicians that had trained in sleight of hand for more than 5 years were able to significantly choose the correct hand when observing a palm transfer technique. This 5-year threshold is worth further exploration: one might wonder whether a magician must have mastered the sleight to be able to recognise it in others, or whether it is a case of having had more experience observing other magicians performing it.

These results are in accordance with most evidence substantiating the level of ability that deliberate practice across time in a particular domain can elicit^50^. However, it should be noted that these skills are not necessarily prompted by the number of years practising per se. Indeed, the opposite might be true in some capacity, the lower performance of individuals with years of experience auditing or performing medical diagnostics when compared to less experienced individuals shows that experience might not necessarily be related to expertise^51^. As such, the expertise skill might be moderated by the type and quality of the practice, rather than the number of years^29^. For example, in singers, amateurs tend to focus on emotional expression amongst other factors when practising, while expert professional singers tend to focus on precision and technique^52^. Such differences in the type of practice might be the cause of the effects we see with magicians when identifying false movements. For example, expert magicians might be focusing more on making their movements as similar to real transfers as possible when practising, and this focus might be granting them the skills necessary to perceive the same technique when performed by others. The positive effect that spending extensive time deliberately practising a skill has on the level of expertise of the individual has been discussed in detail (for a review see^53^). Moreover, even for those with already significant experience, evidence suggests that on average, 10 years practising a particular domain is necessary for both full expert dominion over it, and success at a professional level^54–56^. As such, further experimental evidence should investigate whether the level and quality of the practice that magicians undergo elicit a similar relationship in sleight of hand recognition than the one presented here.

When observing the real palm transfer all participants excelled at identifying the location of the coin regardless of their level of experience practising magic. This was expected as the movement itself reveals the location of the coin. When the experimenter performed the real transfer, the coin was tossed from one hand to the other, thus the observer physically sees the coin move. Moreover, as the movement is a common method of transferring an object from one hand to the other, which is mastered early in child development^57–59^, it is reasonable to assume that any spectator with an unimpaired visual and attentional system would easily locate the hand in which the coin has been transferred to. It is intriguing that, while a very small percentage, some observers in every group still failed to locate the coin. Pperhaps this is just the result of lack of attention when observing the performance, or errors in their response, namely clicking the wrong button by mistake.

In the French drop similar patterns were found to those reported for the Palm transfer in terms of the magic effect condition, namely magicians did, as a group, perform significantly better than non-magicians at identifying the sleight of hand. Non-magicians and magicians with less than 5 years of experience were significantly misled by the French drop technique, while more experienced magicians were able to recognise that the motion presented was mimed, and thus not a real transfer of object.

Regarding the real transfer counterpart od the French drop, non-magicians and magicians with little experience excelled at significantly choosing the correct hand, whilst there was no significant difference in the choices of experienced magicians. These results stand in contrast to the pattern found in the real transfer counterpart of the Palm transfer, in which all participants (including experienced magicians) significantly chose the correct hand. To further explain these results, it is important to reiterate that there is a clear difference between the techniques used in the Palm transfer 1 and in the French drop, not only methodologically but also regarding the amount of perceptual information that the techniques provide to the observer. In the palming technique, whilst there is a simulation of a normal transfer of objects, the movement is not ambiguous (i.e., with attentive observation, the observer can identify that the hand mimicking the transfer is not actually transferring the coin). Similarly, the real transfer counterpart of the Palm transfer offers visual clues to the attentive observer indicating that a real transfer has occurred (such as the visible drop of the coin from one hand to the other). This is not the case for either condition related to the French drop, in which the index and middle finger fully cover the coin (Fig. 1). As the coin is fully covered for both the initial grab of the coin and the retention of it, the observer cannot be certain whether the transfer of objects is real or not (as the coin cannot be seen either way).

The performance exhibited by experienced magicians in the real transfer counterpart of the French drop may be explained by the magician’s mindset rather than their discriminatory ability. Given the ambiguity of the movements observed, it is possible that magicians with several years practising how to fool others by utilising sleight of hand have a more pessimistic mindset than non and less experienced magicians when a motion does not provide clear and observable information. When observing an ambiguous movement, experienced magicians might consider the possibility of foul play and choose accordingly, whilst non-magicians and less experience magicians might not suffer from similar pessimistic perceptive biases. The superior ability of magicians to detect mimed movements from real ones has already been documented by Quarona et al.^40^, where magicians performed significantly better when discriminating the mimed motion of an individual reaching to grasp a glass than non-magicians. However, given our results, this superior ability seems to be developed after several years of training in the craft. Moreover, as evidenced by the experienced magicians' poor performance in the real French drop transfer, this prowess does not seem to grant the experienced magicians with a superior ability to discriminate between mimed and non-mimed movements when such movements are perceptually ambiguous. It is important to note that the magician sample reported in Quarona et al.^40^ had more than 10 years’ experience practicing magic, like the expert magicians reported in this manuscript. However, when observing real reach to grasp actions towards an occluded object, there was no significant difference between magicians and naïve observers in Quarona et al.^40^. This is in contrast to our results, in which we found a significant difference between expert magicians and naïve observers in the real transfer of the ambiguous French drop. It is important to remark that the movements (whether real or pantomimed) displayed by Quarona et al.^40^ were not magic techniques. Consequently, it is possible that this negative bias elicited by our expert sample when observing ambiguous real an ambiguous movement per se. Further evidence is needed to explore whether this is the case.

It is important to note that, in this study, we took a broad-brush approach to magicians’ expertise, and as such the 5-year threshold presented ought to be appraised with caution. Given that the experience level of the participants in this study was measured as four broad groups, the data does not offer insight into the experience variation within the groups (i.e., the exact number of years practising magic), or other variables that might have contributed to such differences in responses (such as the amount of time spent per day practising for example). Consequently, further research is merited to provide insight into the relationship between the number of years of deliberate practice of magicians, and their ability to discriminate deceptive movements. It is worth mentioning that these results could also be influenced by the experimenter performing the sleights of hand. Indeed, evidence suggests that individual motor styles can influence observers’ perception of actions^60,61^. and therefore further tests with different magicians are warranted to fully solidify the responses presented here.

Taken together, our results suggest that whilst, as a group, magicians are better at perceiving deceptive motions in contrast to non-magicians, this ability appears to be moderated by the quantity of experience practising magic, with expert magicians excelling at the recognition of deceptive movements, and non-magicians and magicians with little experience being unable to recognise the fake transfers as deceptive actions. Interestingly the ability to distinguish mimed from real movements does not seem to grant the expert magicians any advantage when these motions are ambiguous in nature. In this case, non-magicians and less experienced magicians seem to be more likely to be correct. We suggest that this might be because expert magicians who have heavily trained in the art of deception, and routinely use their movements to mislead a naïve audience, tend to adopt a pessimistic approach when a movement does not offer clear perceptive clues and consequently, they are more prone to suspect foul play even if the movements are genuine.

## Supplementary Information

Supplementary Video 1.
